# Detecting Methemoglobinemia in Animals with a Drop of Blood

**DOI:** 10.1371/journal.pone.0167942

**Published:** 2016-12-08

**Authors:** Toni G. Patton, Stephen L. Blamer, Katherine E. Horak

**Affiliations:** 1 Fertility Control Project^1^, National Wildlife Research Center, Animal and Plant Health Inspection Service, United States Department of Agriculture, Ft. Collins, Colorado, United States of America; 2 Chemistry Laboratory Unit^2^, National Wildlife Research Center, Animal and Plant Health Inspection Service, United States Department of Agriculture, Ft. Collins, Colorado, United States of America; US Geological Survey, UNITED STATES

## Abstract

A major concern during pesticide development and use is the impact on non-target species, such as raptors or domestic cats and dogs. Sodium nitrite and para-aminopropiophenone (PAPP) are two toxicants currently being studied for the control of invasive species, such as starlings and feral swine. When given to an animal these compounds oxidize hemoglobin, which renders it unable to carry oxygen resulting in methemoglobinemia. This study developed a method to estimate methemoglobin levels in mammals and birds by examining the efficacy of sodium nitrite to induce the conversion of hemoglobin to methemoglobin. Varying concentrations of sodium nitrite were added to aliquots of coyote, vole, feral swine, starling, and duck blood, collected from captive animals. The blood samples were analyzed spectrophotometrically to determine percent methemoglobin and digitally to determine red color values (RCV) associated with different methemoglobin levels. The avian and mammalian blood reached 100% methemoglobin levels at 200 mM and 15 mM sodium nitrite, respectively. All animals had similar RCV for a given percent methemoglobin. In conclusion, this study developed a procedure to quickly determine methemoglobin levels in mammals and birds. Furthermore, percent methemoglobin can be estimated with one standard curve from any animal species and an image of a blood spot. The technique will be useful during field studies, in agricultural areas, or in a veterinarian’s office for the rapid diagnosis of methemoglobinemia in non-target animals that have eaten toxicants/baits or baited animals.

## Introduction

Invasive species, like starlings and feral swine, inflict extensive damage to ecosystems worldwide and are a growing concern to agricultural industries [1–4]. Many currently used pesticides are costly and are becoming less effective for controlling pests. Furthermore, these compounds are coming under scrutiny related to their mechanisms of action and potential risks to non-target species, like raptors or domestic cats and dogs [1, 5–10]. Several studies have examined methemoglobin-inducing agents to control invasive species populations because some of these compounds, such as para-aminopropiophenone (PAPP) and sodium nitrite, have antidotes [1, 8–17] [18].

Methemoglobin is formed by the oxidation of the iron atom in hemoglobin from its ferrous to ferric form [17, 18]. The oxidation reaction impairs hemoglobin’s ability to transport oxygen, leading to tissue hypoxia and possibly death [18]. A small percentage of methemoglobin is commonly found in the blood, but can increase due to genetic disorders, diet, injury, or toxins [17, 19]. Few clinical signs of methemoglobinemia are exhibited when the proportion of methemoglobin to total hemoglobin is below 10%, but levels above 10% can cause skin discoloration, frequent urination, and restlessness [18, 20]. Furthermore, methemoglobin levels above 50% have been shown to cause seizures, comas, or death [17, 18, 20].

Hemoglobin has a deep red color and methemoglobin is dark chocolate brown. This color difference can be exploited to calorimetrically measure the relative proportion of methemoglobin to total hemoglobin in a blood sample [18, 20, 21]. Methemoglobin can also be measured spectrophotometrically using an absorption peak of 635 nm [21–23]. As compared to hemoglobin with two absorption peaks at 540 and 580 nm [21–24]. These two absorption peaks diminish and the 635 nm methemoglobin peak develops upon addition of hemoglobin oxidizing compounds to blood samples [24].

Sodium nitrite is an inorganic salt that directly oxidizes hemoglobin and is a strong inducer of methemoglobinemia [25–27]. This chemical is a good candidate for *in-vitro* efficacy studies because it is direct acting and there is a linear relationship between sodium nitrite concentration and methemoglobin formation [21, 23, 28]. In this study, we utilized sodium nitrite, the 635 nm methemoglobin absorption, and the characteristic color change to develop a colorimetric technique and color cards to estimate methemoglobin levels of birds and mammals exposed to hemoglobin oxidizing compounds.

## Materials and Methods

### Ethics statement

Blood was collected from captive Eastern European starlings (*Sturnus vulgaris*), mallard ducks (*Anas platyrhynchos*), feral swine (*Sus scrofa*), California voles (*Microtus californicus*), and coyotes (*Canis latrans*) in accordance to approved United States Department of Agriculture, National Wildlife Research Center (NWRC) or Colorado State University IACUC protocols (QA2177, QA2290, 14-5367A, QA2114, and QA2346). All blood draws were performed at the NWRC in a necropsy room or animal pens as described in the IACUC-approved protocols and all efforts were made to minimize suffering of all captive animals.

### Blood processing

Fresh whole blood samples from captive starlings (N = 9), ducks (N = 4), feral swine (N = 3), voles (N = 3), and coyotes (N = 3) were processed in the laboratory using a modified method previously described by Martinez-Haro and Mateo [23]. Briefly, 45 μl of blood per animal was aliquoted into individual microcentrifuge tubes, and 5 μl of phosphate buffered saline (PBS) or sodium nitrite solution, at increasing concentrations, were added to the blood samples. The samples were mixed and incubated at room temperature for two minutes. Following the incubation, blood samples were diluted (1:100) and lysed with water, mixed by inversion, and incubated for two minutes at room temperature. The lysed mammalian blood (non-nucleated) samples were clear and processed directly without centrifugation. However, the nucleated avian blood samples were turbid and centrifuged for four minutes at 3,500 X g and 4°C (Legend Micro 21R centrifuge, Thermo Scientific, Marietta, OH). The resulting supernatant was transferred to clean microcentrifuge tubes and analyzed.

### Photometric spectrum scans

To verify that the blood samples possessed the characteristic absorption peaks at 540, 580, and 635 nm we added 200 μl of red blood cell lysates, in triplicate, to 96 well plates and scanned using a Varioskan Flash (Thermo Scientific, Marietta, OH). Photometric spectrum scans were performed for each blood sample from 400 nm to 700 nm with a 10 nm step size and a 100 ms measurement time at room temperature.

### Spectrophotometric analysis

Percent methemoglobin was determined spectrophotometrically (Orion Star A, Thermo Scientific) as described previously by Martinez-Haro and Mateo [23]. Briefly, fresh neutralized potassium cyanide (KCN; Sigma-Aldrich, St. Louis, MO) solution was prepared by combining one part 4.07% KCN with one part 4.88% acetic acid (Fisher, Pittsburgh, PA); this solution was used within one hour of preparation. Eight hundred and eighty microliters of phosphate buffer (A; 9.85 mM KH2PO4, 5.26mM Na2HPO4, pH 6.6; Sigma-Aldrich) or 0.06% potassium ferricyanide (B; Sigma-Aldrich) was aliquoted into cuvettes followed by the addition of 80 μl of lysed red blood cells. The cuvette was mixed by inversion and the absorbance at 635 nm was recorded (A1 or B1). Then 20 μl of neutralized KCN was added to the cuvettes, mixed by inversion, and an additional optical density at 635 nm was documented (A2_1_ or B2_1_). After a two minute room temperature incubation another absorbance at 635 nm was recorded (A2_2_ or B2_2_). This was performed for each lysed blood sample in phosphate buffer (A) and potassium ferricyanide (B). Percent methemoglobin was calculated with the following equation: [(A1-A2)/(B1-B2)]X100, for both the initial and two minute post KCN addition readings.

### Colorimetric assay

A colorimetric assay was performed with the sodium nitrite treated blood using a modified method previously described by Shihana et al [21]. Briefly, 10 μl of PBS or sodium nitrite treated blood was spotted onto Whatman 40 ashless filter paper (GE Healthcare Life Sciences, Pittsburgh, PA). The blood-spotted filter paper was scanned immediately using a flatbed scanner (Sano Scanlide 110 U1.2, Canon, Melville, NY or Epson Perfection 1640OSU, Long Beach, CA). The generated Tiff images were analyzed using ImageJ software (http://imagej.nig.gov/ij/). Red (RCV), blue (BCV), and green (GCV) color values were determined for five sections, selected with ImageJ’s point tool, per blood spot and averaged. Red color value was the only predictive parameter, therefore the average RCV were regressed against percent methemoglobin values determined using a spectrophotometer. A standard curve was generated from this regression.

This standard curve was used to compare RCV of blood samples with known methemoglobin concentrations to validate the colorimetric analysis methods for predicting percent methemoglobin. The predictive power of the colorimetric analysis was determined using a repeated measures regression performed with RCV as the explanatory variable (Y). The generated linear models were for individual species and all species combined. The predictive power for the generated species specific and all species models was calculated using SAS 9.4 (SAS Institute, Inc., Cary, NC, USA)

## Results

**Fig 1:**
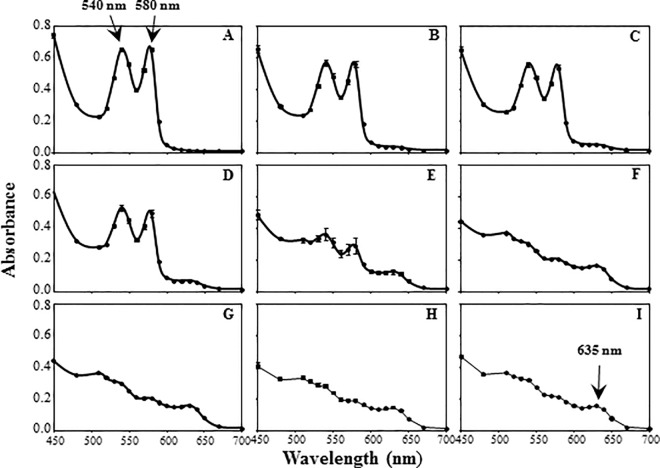
Representative spectral scan of lysed blood cells with increasing concentrations of sodium nitrite. (A) 0 mM, (B) 6 mM, (C) 12 mM, (D) 15 mM, (E) 19 mM, (F) 25 mM, (G) 29 mM, (H) 58 mM, (I) 102 mM of sodium nitrite was added to blood aliquots, lysed, and added to 96 well plates in triplicate. Optical density was measured from 400 nm to 700 nm for each well. Arrows point to the hemoglobin peaks at 540 and 580 nm and a methemoglobin peak at 635 nm. Error bars correspond to the standard errors of the means.

**Fig 2:**
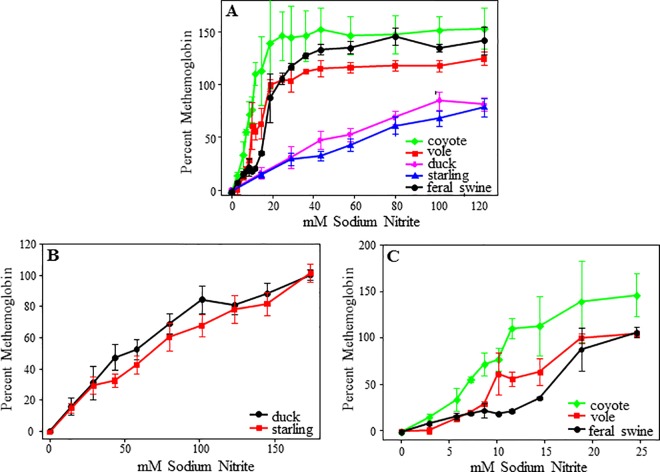
Methemoglobin levels in blood samples exposed to increasing concentrations of sodium nitrite. (A) All species, (B) Avian blood samples, and (C) Mammalian blood samples. The data reported are the initial post KCN addition optical densities and represent the average of 3 (mammals), 4 (duck), or 9 (starlings) independent blood samples exposed to PBS or sodium nitrite. Error bars correspond to the standard errors of the means.

As seen in Fig 1. the blood samples had the two characteristic hemoglobin peaks at 540 and 580 nm prior to the addition of sodium nitrite (Fig 1). These two hemoglobin peaks were reduced with increasing concentrations of sodium nitrite (Fig 1). Furthermore, a small peak at 635 nm, representing methemoglobin, began to form at higher sodium nitrite concentrations (Fig 1). These results were consistent across species with all blood samples exhibiting peaks at the same wavelengths. The peak at 635 nm, characteristic of methemoglobin, formed at lower sodium nitrite concentrations in the mammalian samples than in the bird samples. This difference was further observed with the spectrophotometric analysis, revealing that the avian samples reached 100% methemoglobin near 200 mM sodium nitrite and the mammalian blood reached 100% methemoglobin at approximately 15 mM sodium nitrite (Fig 2).

**Fig 3:**
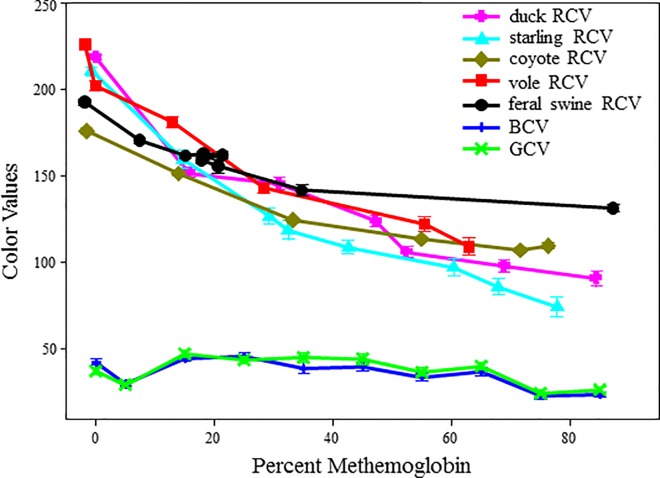
Red color value variation with increasing methemoglobin levels. Blood samples were treated with sodium nitrite or PBS, spotted onto Whatman filter paper, and scanned with a flatbed scanner. Scanned images were analyzed with ImageJ software to generate RCV, GCV, and BCV. The RCV data presented represents the average of 3 (mammal), 4 (duck), or 9 (starling) different scanned images and the error bars correspond to the standard errors of the means. GCV and BCV data represents an average of all 19 blood spot images from all species.

Upon addition of sodium nitrite, the red colored hemoglobin was oxidized forming the characteristic brown colored methemoglobin in all animals tested. As methemoglobin levels increased, RCV decreased, but the blue and green color values remained the same (Fig 3). The vole, duck, and starling blood spot images had RCV over 200 at 0% methemoglobin and were not statistically significant from each other (P < 0.05; Fig 3). The 0% methemoglobin RCV for the coyote (176) and the swine (191) blood samples were statistically significant from the vole, duck, and starling (P > 0.05). Despite the difference at 0% the RCV were statistically similar between 10–50% methemoglobin for all species (Fig 3). Accounting for all the species the repeated measures regression model showed that the species’ RCV were not significantly different from each other (p < 0.001).

The standard curve equations and random blood spot images were used to predict methemoglobin levels to ascertain the predictive power of the colorimetric model compared to the spectrophotometric assay (Table 1). As seen in Table 1, the median colorimetric predicted percent methemoglobin values are within 8.6% of the spectrophotometric values.

**Table 1:** Comparative analysis of the spectrophotometric and colorimetric predicted percent methemoglobin levels.

Species		General Model	Individual Model
All Species	Average	10.08	
	Median	8.59	
Eastern European Starling	Average	13.37	10.02
	Median	11.77	8.64
Mallard Duck	Average	9.08	9.08
	Median	8.36	8.36
Feral Swine	Average	10.9	11.17
	Median	7.32	7.73
California Vole	Average	6.97	6.84
	Median	5.42	5.54
Coyote	Average	8.26	14.9
	Median	7.17	16.49

The differences in percent methemoglobin were calculated using general (all species) and individual predictive models.

## Discussion

The impact on non-target species, like domestic cats and dogs or raptors, which may eat either the toxicant/bait or baited animals is a concern with current pesticide use and development. Therefore, it is important to have a method to determine potential intoxications of non-target species so antidotes can be administered as soon as possible. In this study, we examined the efficacy of a strong hemoglobin oxidizing compound, sodium nitrite, to estimate methemoglobin levels in mammalian and avian blood samples [21, 23]. We then developed a technique that can be used in veterinarian’s offices, in agricultural areas, or during field trials to estimate methemoglobin levels in mammals and birds.

Interestingly, the mammalian and avian blood appeared to respond differently to challenge by sodium nitrite (Fig 2A). We observed that the mammalian blood samples oxidized at lower concentrations of sodium nitrite than the avian blood samples (Fig 2A). The sensitivity differences may be due to the nucleated red blood cells and other structural differences in the avian hemoglobin that are not present in mammalian blood [29]. Although there were differences in the effect of sodium nitrite on methemoglobin formation between mammals and birds, the relationship of sodium nitrite concentration to hemoglobin formation was linear regardless of species (Fig 2B and 2C).

**Fig 4:**
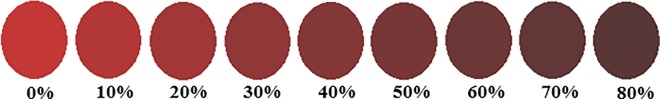
Percent methemoglobin color chart. Predicted RCV generated from the starling, duck, and vole’s standard curve equations were averaged and used to generate custom color cards in MS Paint. Blue and green color values were estimated to be 40, which was based on the average blue and green color values in these samples.

With this analysis we developed a color card and a colorimetric assay from images of freshly spotted blood (Figs 3 and 4). This colorimetric scale utilized the red to brown color change that occurs as hemoglobin is oxidized into methemoglobin [18, 20, 21]. Red, blue, and green color values were measured in all the blood samples, but only the red color value had predictive power for estimating percent methemoglobin. All the animals showed comparable RCV at similar methemoglobin levels, except the coyote and feral swine at 0% methemoglobin (Fig 3). The feral swine and coyote blood samples had significantly reduced RCV at 0% methemoglobin compared to the other three species (P > 0.05). This discrepancy may be due to a different scanner that was used, which did not produce as crisp of images as the scanner used for the voles, starlings, and ducks. Therefore, to ensure consistent results the digital image should be generated from the same flatbed scanner or camera for all samples. It is also important to use fresh uncoagulated blood to develop the standard curves. One day old refrigerated whole blood or coagulated blood produced altered standard curves for both the spectrophotometric and colorimetric analysis when compared to fresh blood samples (data not shown). Furthermore, neither method was very sensitive to extremely low (less than 5%) nor high amounts of methemoglobin (over 70%). The sensitivity of either test is of little concern at the miniscule amounts of methemoglobin because the risks associated with these low methemoglobin levels are minimal. The lack of sensitivity over 70% may be a drawback of this assay if an accurate methemoglobin level is necessary to determine cause of death or lethal methemoglobin levels.

Consistency between the two methods, spectrophotometric and photometric, decreased as the percent methemoglobin levels increased (data not shown). This is possibly due decreased sensitivity of the ImageJ software as the blood gets darker and/or because of non-uniform color across an entire blood spot. The filter paper was scanned immediately after applying the blood samples; however we still observed coloration differences in some individual blood spots (data not shown).

In the field of wildlife toxicology, many studies are performed outdoors without access to laboratory instrumentation. Therefore, it was necessary to develop a method to estimate methemoglobin levels that would generate consistent results and could be performed onsite with a color card or by colorimetric analysis of a digital image sent to a colleague with access to a computer (Fig 4). To determine the predictive power of the colorimetric analysis we compared predicted methemoglobin values, calculated using a repeated measures regression model for individual and all species combined, to the percent methemoglobin levels determined spectrophotometrically (Table 1). The absolute value of the differences suggests that the predicted colorimetric value would be within 8.6% of what would be generated with a spectrophotometer (Table 1). Therefore, percent methemoglobin can be estimated within minutes of exposure to methemoglobin forming compounds with a drop of blood spotted on white paper. Not only will this be useful in field studies, but farmers that deploy methemoglobin inducing toxicants can rapidly diagnose methemoglobinemia with a color card or picture taken with a cell phone. The picture can be sent to someone with a computer for quick analysis. After the rapid diagnosis the farmer can either administer an antidote to exposed animals or take the animal to a veterinarian’s office for treatment. A similar technique has been successfully applied in a clinical setting resulting in improved treatment, and therefore decreased human fatalities [30–32]. This indicates that applying our technique to mammals and birds could also result in decreased fatalities.

In this study, we provide a simple method to determine percent methemoglobin in animals that requires a small amount of fresh blood. Furthermore, we demonstrated that with one standard curve and a blood spot image percent methemoglobin can be estimated in a timely fashion. If an image cannot be obtained and an immediate approximation is necessary, a color card was also created for comparison of a blood spot to a color standard (Fig 4). Both the color card and the colorimetric assay will be useful for the diagnosis of methemoglobinemia in non-target species. Through the use of these field or veterinary office ready techniques, this study will assist in the protection of non-target species that have been exposed to hemoglobin oxidizing compounds.
